# A rare presentation of the diffuse dermal mucinosis in adenocarcinoma of the lungs treated with pembrolizumab and pemetrexed

**DOI:** 10.1016/j.jdcr.2025.04.014

**Published:** 2025-04-27

**Authors:** Karen Koch, Kamal Solanki, Julia Zhu

**Affiliations:** a Te Whatu Ora Health Waikato, Waikato Hospital, Hamilton, New Zealand; b Waikato School of Medicine, Auckland University, Hamilton, New Zealand; c Apollo Hospitals Educational and Research Foundation, Hyderabad, India

**Keywords:** drug-related mucinosis, generalized mucinosis, mucinosis, paraneoplastic mucinosis, pembrolizumab, pemetrexed, scleredema

## Introduction

Mucinosis are a rare group of disorders characterized by accumulation of mucin in the skin and other organs. Subtypes include lichen myxedematosus (scleromyxedema and localized forms), scleredema, thyroid disease-related, reticular erythematous mucinosis, papulonodular mucinosis associated with connective tissue diseases, and cutaneous focal mucinosis.^1^ More recently emerging subtypes have been recognized secondary to lymphedema of obesity, pretibial stasis, medications, and mechanical trauma.^1^

We present a 69-year-old woman with diffuse dermal mucinosis with onset 3 months after her diagnosis of right upper lobe stage IIIC (cT3 cN3 cMx) adenocarcinoma and 1 month after initiation of carboplatin, pemetrexed, and pembrolizumab. Symptoms continued to progress on pemetrexed and pembrolizumab and spontaneously improved on stopping treatment. This unusual presentation may indicate a new form of drug-related mucinosis (due to pemetrexed and/or pembrolizumab) or extensive scleredema-like paraneoplastic mucinosis.

## Case report

**Fig 1:**
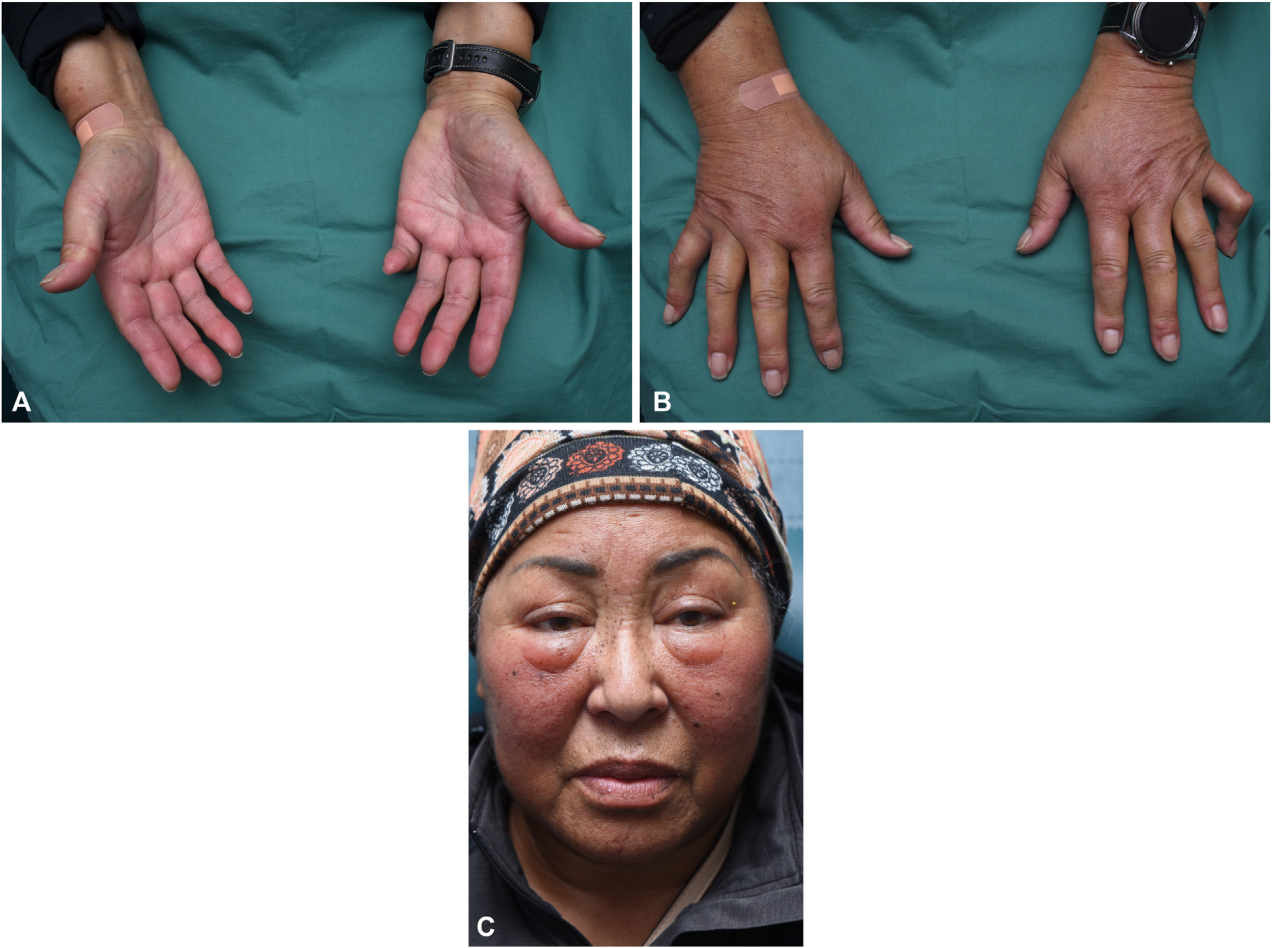
Photos showing the swelling of face and hands. **A,** Swelling of the dorsum aspect of the hands. **B,** Swelling of the palmar aspect of the hands. **C,** Swelling of the face.

A 69-year-old woman presented with an 11-month history of diffuse skin thickening of her face, arms, legs, hands, and feet (Fig 1, *A-C*). Initial skin symptoms started as nonpitting swelling and erythema on the face 1 month after initiation of chemotherapy for right upper lobe stage IIIC (cT3 cN3 cMx) adenocarcinoma (K-ras mutated, EGFR/BRAF negative). Her lung cancer was initially treated carboplatin/pemetrexed + pembrolizumab × 4 cycles, followed by monthly pemetrexed and pembrolizumab (31 cycles). Her lung cancer responded well to chemotherapy within the first 4 months of therapy and has remained stable.

She is a hepatitis B carrier (hepatitis B surface antigen positive and hepatitis B e antigen negative) under long-term liver surveillance (but no active therapy) for the last 11 years. Her viral load before chemotherapy was 1402 IU/mL and she remained on entecavir throughout her chemotherapy (viral load of 308 IU/mL during this period).

She has a background of hypertension, atrial fibrillation, hearing impairment and is a previous smoker (20-year pack history).

**Fig 2:**
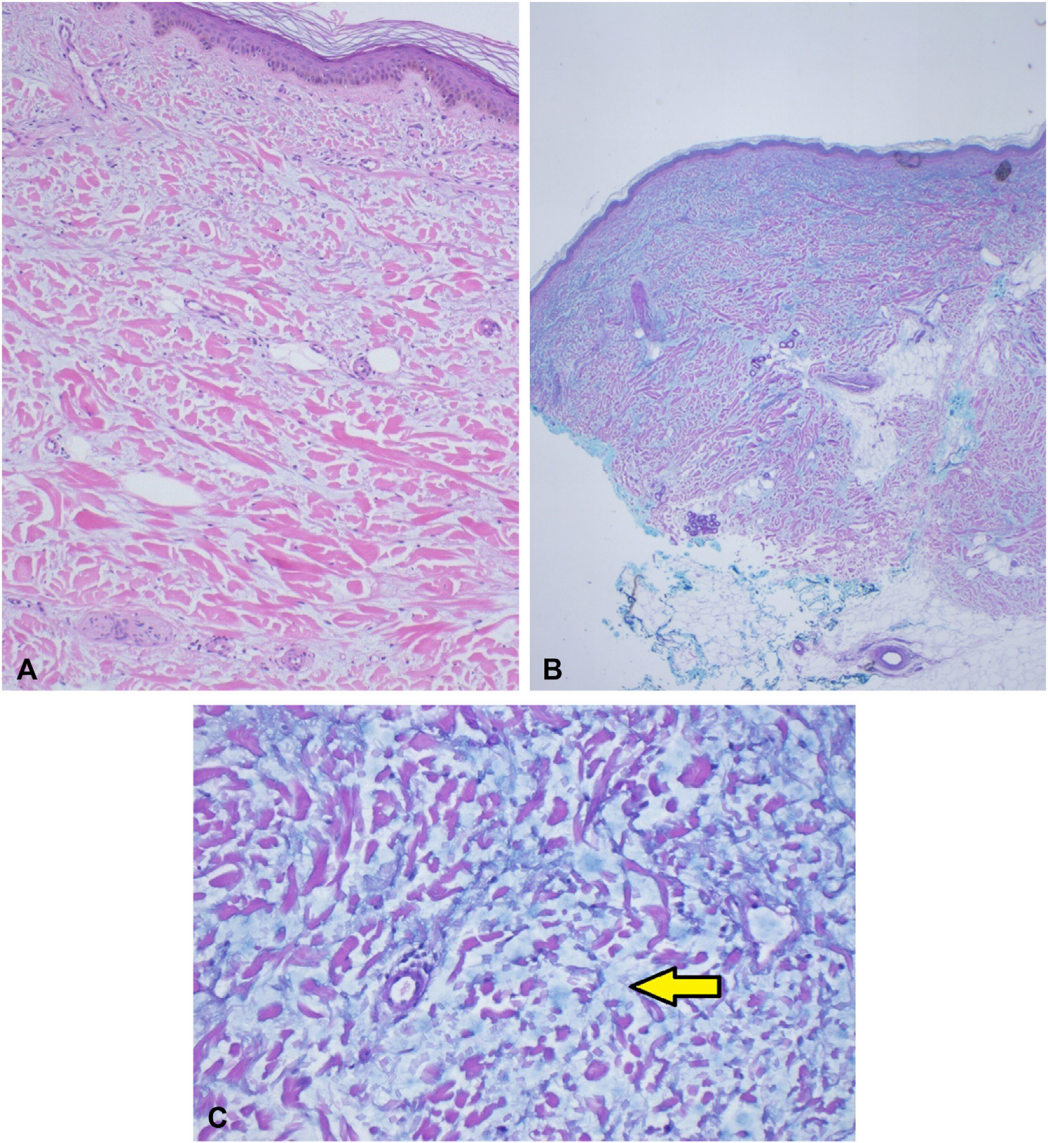
**A,** Skin biopsy with hematoxylin-eosin stain, demonstrating normal epidermis. **B, C,** Skin biopsy with Alcian blue and periodic acid–Schiff stains. The increased blue material between the collagen bundles is mucin (see *arrow*). (**A,** Hematoxylin-eosin stain; **B,** Alcian blue stain; **C,** periodic acid–Schiff stain; original magnifications: **A,** ×100; **B,** ×20; **C,** ×200.)

Incisional biopsies of the upper portion of the right arm and anterior aspect of left thigh (12 months after the initiation of chemotherapy) showed diffuse interstitial dermal infiltration of mucin with minimal inflammation (Fig 2, *A-C*). Repeat biopsies of the upper portion of the right arm and anterior aspect of left thigh (7 months after completing chemotherapy) showed significant reduction in mucin.

Serum protein electrophoresis was normal on repeated testing, with no paraproteins detected. She has had a single raised kappa light chain result (34.1 mg/L; normal range, 3.3-19.4 mg/L) with a preserved kappa:lamba ratio. Repeated diabetes and thyroid screening have been unremarkable. She had a full rheumatological work-up with a negative antinuclear antibody and scleroderma panel. She has had no eosinophilia on full blood count. She had mild chemo-related lymphopenia (0.6 × 10E9/L).

**Fig 3:**
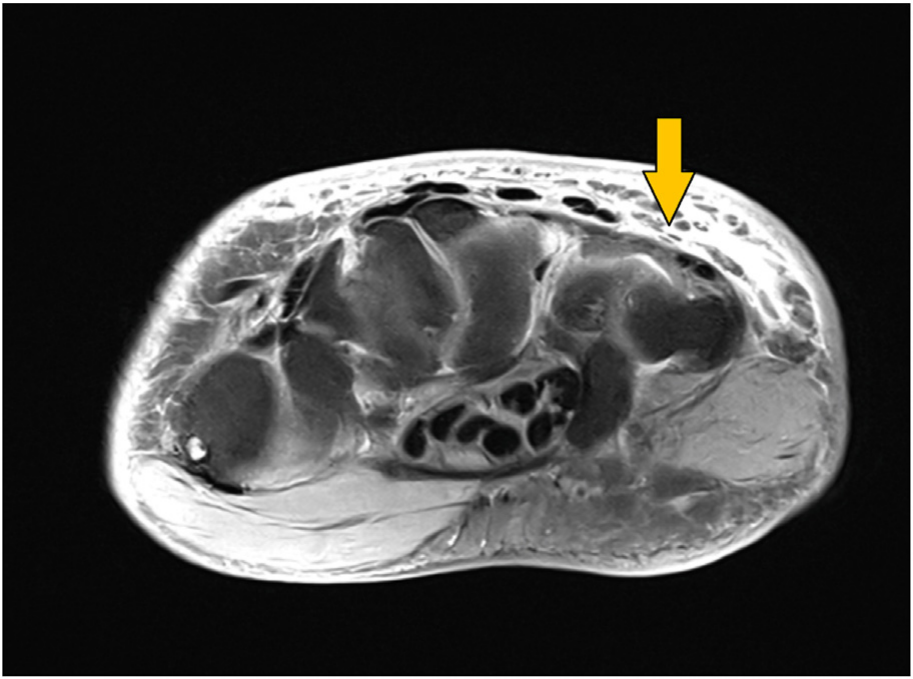
Magnetic resonance imaging of the right hand during treatment (T2 coronal view): *yellow arrow* pointing to the subcutaneous mucinosis.

Cardiac magnetic resonance imaging showed mild cardiomyopathy but no infiltrative disease. Magnetic resonance imaging (18 months after the onset of symptoms) demonstrated subcutaneous thickening of the dermis and subcutis of the wrist, dorsal surfaces of the hands and feet as well as periorbital soft tissue causing symmetric proptosis (Fig 3).

Given the risk of systemic corticosteroids reducing the efficacy of her pembrolizumab, we opted for whole body narrowband UV-B for 3 months (29 sessions) and psoralens UV-A of the hands and feet for 5 months (19 sessions). She had no improvement with narrowband UV-B but some response to psoralens UV-A. Unfortunately, treatment was interrupted with bouts of illness related to her chemo.

Consideration was given to stopping her pembrolizumab and pemetrexed, but her lung cancer was stable on treatment and alternative options were limited. We could not entirely exclude the condition as being paraneoplastic rather than medication-induced (although her skin disease worsened while her lung cancer remained radiologically unchanged). The risks of continuing chemotherapy were deemed preferable to stopping treatment. We considered intravenous immunoglobulin but delayed treatment as she was nearing completion of her chemotherapy.

**Fig 4:**
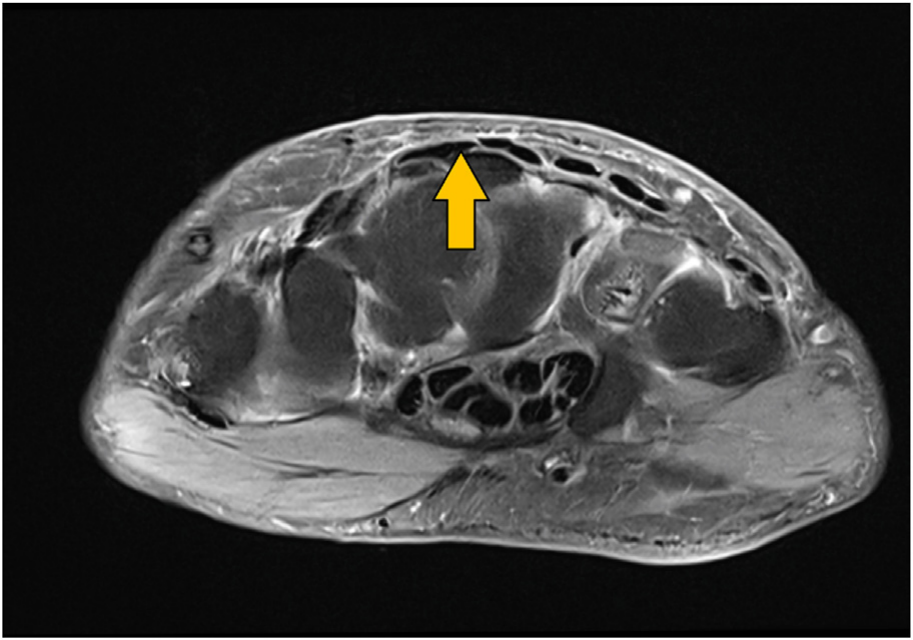
Magnetic resonance imaging of the right hand 8 months after completing chemotherapy showing reduction in dermal mucin (*yellow arrow*).

Since completing her chemotherapy, her skin symptoms have improved. We confirmed this with repeat magnetic resonance imaging (8 months after stopping chemo) (Fig 4) and skin biopsies (7 months after stopping chemotherapy). She has persistent facial swelling with thickened subcutaneous tissue in the upper cheeks and mid-forehead. She has residual thickening on her lateral aspect of upper thighs and to a lesser extent on her bilateral upper arms. Her lung cancer has remained stable. We continue to monitor her for hematologic malignancies.

## Discussion

Our patient presented without pitting edema and her initial swelling involved her face. She had no fluctuation in symptoms but rather steady progression. She had no arthralgia but developed joint stiffness as her condition worsened. She had diffuse dermal mucin deposition without waxy papules, clinically resembling scleredema. Scleredema typically involves the mid-upper back, neck, and shoulders, but some case reports have described involvement of the face, mouth, esophagus, and the heart.^2^ Involvement of the bilateral hands and feet, forearms, and lower portion of the legs has not been widely reported.

In adults, scleredema is usually related to diabetes or other underlying conditions (febrile/infection-related and monoclonal gammopathy-related).^3^ It has been reported secondary to hepatitis B.^4^ We considered this as a differential diagnosis, but her viral load did not increase during chemotherapy and her symptoms have continued to improve, in spite of a slight increase in her viral load after stopping entecavir.

The generalized smooth appearance of her mucinosis was not consistent with scleromyxedema and we excluded a monoclonal gammopathy.^5^

Pembrolizumab can cause edema and generally involves the hands, feet, and lower limbs.^6^ Remitting seronegative symmetric synovitis with pitting edema syndrome is a rare entity causing pitting edema of hands and feet and has been associated with both lung cancer and pembrolizumab.^7^ Facial and generalized edema, as well as pseudocellulitis has been reported with the use of pemetrexed as a single agent, as well as in combination with pembrolizumab.^8^ The finding of mucin deposition excluded edema as the primary pathogenesis.

Pembrolizumab-induced cutaneous lupus is a reported phenomenon and lupus can present with periorbital mucinosis deposition.^9^ Our patient had no other histological, laboratory, or clinical findings to suggest lupus.

Paraneoplastic morphea with increased mucin deposition has been reported in a patient with smoldering myeloma.^10^ Our patient had no collagen changes (sclerosis or hyalinization) on histology to suggest morphea.

Drug-related mucinosis has been linked to interferon alfa-1, interferon beta-1, interleukin 12/23 inhibitors (ustekinumab), tumor necrosis factor-alfa inhibitors and interleukin 6 inhibitors (tocilizumab). These tend to be more localized, and none reported diffuse whole-body involvement.^1^ Diffuse dermal mucinosis has been documented in 4 patients treated with anti–colony-stimulating factor 1 receptor medication in the form of waxy pseudoepitheliomatous skin changes with a “cobblestone” appearance.^11^

In conclusion we report a patient with an unusual diffuse scleredema-like cutaneous mucinosis that may be drug-induced or paraneoplastic in origin. It does not fit well into any current classification of mucinosis.

## Conflicts of interest

Dr Solanki is an Honorary Adjunct Professor of Apollo Hospitals Educational and Research Foundation (AHERF), India. Drs Koch and Zhu have no conflicts of interest to declare.
